# Global burden of thyroid cancer in males: a comprehensive analysis of incidence, mortality, and risk factors from 1990 to 2040

**DOI:** 10.3389/fonc.2026.1699986

**Published:** 2026-02-19

**Authors:** Hao Chen

**Affiliations:** Department of Surgical Oncology, The First Affiliated Hospital of Xi’an Jiaotong University, Xi’an, China

**Keywords:** disease trends, gender disparities, global burden, risk factors, thyroid cancer

## Abstract

**Background:**

Thyroid cancer incidence has increased globally, with notable gender disparities in epidemiological patterns and outcomes. This study examines the global burden of thyroid cancer in males from 1990 to 2021 and projects trends to 2040.

**Methods:**

Using data from the Global Burden of Disease Study 2021, the author analyzed age-standardized incidence, prevalence, deaths, and disability-adjusted life years (DALYs) for thyroid cancer in males across 204 countries and territories. Bayesian meta-regression and ensemble modeling techniques were employed to estimate trends and generate projections.

**Results:**

Global male thyroid cancer incidence increased from 1.2 to 2.0 per 100,000 (EAPC: 1.78%, 95% CI: 1.64-1.92) between 1990 and 2021, while mortality remained stable (0.4 to 0.5 per 100,000; EAPC: 0.41%, 95% CI: 0.35-0.46). Significant geographical variations were observed, with high-SDI regions showing the highest incidence rates but the most stable mortality patterns. Projections to 2040 indicate continued incidence increases (2.16 per 100,000) with stable mortality (0.45 per 100,000), though uncertainty intervals widen substantially in later years.

**Conclusion:**

The diverging trends between rising incidence and stable mortality in male thyroid cancer suggest substantial, particularly in developing regions. Gender differences in disease characteristics and outcomes highlight the need for sex-specific clinical approaches. These findings emphasize the importance of implementing evidence-based diagnostic practices to minimize overdiagnosis while ensuring appropriate care for advanced cases.

## 1 Introduction

Thyroid cancer represents the most common endocrine malignancy, with a rapidly increasing global incidence that has nearly tripled over the past three decades (1–3). While historically considered a disease predominantly affecting women, emerging evidence reveals significant gender disparities in epidemiological patterns, clinical presentation, and therapeutic outcomes (4, 5). The female-to-male incidence ratio of approximately 3:1 mask important difference in disease characteristics and temporal trends that warrant detailed investigation (4, 6).

The global burden of thyroid cancer exhibits substantial geographical variation, with higher incidence rates typically observed in high-income countries, potentially reflecting more intensive diagnostic practices (7). However, the drivers behind the disproportionate increase in male thyroid cancer cases in many regions remain poorly understood (8). Previous studies have suggested that biological factors, including hormonal influences and genetic susceptibility, may contribute to these gender differences (9, 10). Additionally, variations in healthcare-seeking behavior, occupational exposures, and lifestyle factors may further complicate the gender-specific epidemiology of thyroid cancer (11, 12).

Despite the lower absolute incidence in males, accumulating evidence indicates more aggressive clinicopathological features and worse survival outcomes in male patients compared to females (13, 14). Male patients typically present with larger tumors, higher rates of extrathyroidal extension, and more advanced disease stage at diagnosis (15, 16). These differences highlight the potential need for gender-specific approaches to screening, diagnosis, and management of thyroid cancer (17).

The Global Burden of Disease (GBD) study provides a comprehensive framework for examining population-level trends in disease burden across demographic groups and geographical regions (18, 19). Previous GBD analyses have documented the increasing global burden of thyroid cancer but have not specifically focused on gender disparities in disease patterns and temporal trends (20, 21). Understanding these differences is crucial for developing targeted prevention strategies and optimizing healthcare resource allocation, particularly in the context of evolving diagnostic practices and risk factor distributions.

This study aims to provide a comprehensive analysis of the global burden of thyroid cancer in males from 1990 to 2021, with projections to 2040, using data from the GBD 2021 study. The author examines gender differences in incidence, prevalence, death, and disability-adjusted life years (DALYs) across different regions and socio-demographic index (SDI) categories. Bayesian Age-Period-Cohort (BAPC) modeling was employed for projections and include validation of forecasting accuracy. Additionally, the author assesses the burden associated with high body-mass index and explore temporal trends to inform evidence-based policies and clinical practices for male thyroid cancer patients worldwide.

## 2 Materials and methods

### 2.1 Data sources

The data for this study were obtained from the Global Burden of Disease Study 2021 (GBD 2021), coordinated by the Institute for Health Metrics and Evaluation (IHME), which provides comprehensive estimates of incidence, prevalence, mortality, and disability-adjusted life years (DALYs) for 369 diseases and injuries in 204 countries and territories from 1990 to 2021 (17). Thyroid cancer cases were identified using International Classification of Diseases (ICD) codes ICD-10 C73 and ICD-9 193. Prevalence estimates accounted for both diagnosed and undiagnosed cases, adjusted for disease remission and competing mortality risks using DisMod-MR 2.1, a Bayesian meta-regression tool. Mortality data were derived from cause-of-death records, with redistribution algorithms applied to correct for ill-defined coding practices.

Countries were classified into five socio-demographic index (SDI) quintiles (low, low-middle, middle, high-middle, high) based on per capita income, educational attainment, and total fertility rate. Additionally, 24 GBD regions were analyzed to capture subglobal epidemiological patterns. Age-specific estimates were generated for 17 age groups (5-year intervals from 15–19 years to ≥95 years), enabling detailed life-course assessments. The analysis focused on the population aged 15 years and older, as thyroid cancer is exceptionally rare in children, to ensure stable and interpretable estimates for the primary at-risk demographic.

### 2.2 Risk factor assessment

The comparative risk assessment framework was employed to estimate the burden of thyroid cancer attributable to high body-mass index (BMI). This analysis focused on high body-mass index (BMI) due to its established association with thyroid cancer and its significance as a major modifiable risk factor with sufficient data for global estimation. High BMI was defined as ≥25 kg/m² for adults, with a theoretical minimum risk exposure level of 20–25 kg/m² based on World Health Organization guidelines. Relative risks for thyroid cancer associated with high BMI were derived from published meta-analyses and pooled cohort studies. Population attributable fractions (PAF) were calculated using the standard formula: $PAF=\frac{\sum Pi\;\left(RRi-1\right)}{\sum Pi\;\left(RRi-1\right)+1}$, where P_i_ is the proportion of the population in exposure group, and RR_i_ is the relative risk for that group.

### 2.3 Forecasting methodology

Projections to 2040 were generated using an ensemble modeling approach. This approach combined three distinct modeling strategies: (1) a simple linear regression model based on recent trends; (2) a component model that projected future changes based on projected SDI trajectories, which serve as a composite proxy for future changes in healthcare access, education, and economic development, including shifts in risk factor prevalences like obesity; and (3) a Gaussian process regression model that incorporated spatiotemporal smoothing and uncertainty in future rates (22). To validate the forecasting approach, the ensemble model was fitted to data from 1990–2015 and used to predict the period 2016-2021. The model performance was quantitatively assessed using the Mean Absolute Percentage Error (MAPE).

### 2.4 Statistical analysis

The analysis employed Cause of Death Ensemble modeling (CODEm) and Bayesian meta-regression techniques to estimate mortality and incidence rates. DisMod-MR 2.1was used to ensure internal consistency between incidence, prevalence, remission, and mortality estimates. Age-standardized rates (ASRs) were calculated using the GBD world standard population to allow for comparison across populations and over time (23). Uncertainty intervals (UIs) were generated using 1000 draws from the posterior distribution of each estimate. The 95% UIs represent the 2.5th and 97.5th percentiles of these ordered draws. Estimated annual percentage changes (EAPCs) were calculated to quantify trends over time using the formula: EAPC = 100 × (exp(β) - 1), where β is the regression coefficient from a linear regression model fitted to the natural logarithm of the annual ASRs (24).


$$ASR=\frac{\sum_{i=1}^{A}a_{i}w_{i}}{\sum_{i=1}^{A}w_{i}}\;\times 100,000$$

The 95% confidence intervals for EAPCs were derived from the linear regression model. Analyses were executed using R 4.2.0 for statistical modeling. As the study relied exclusively on anonymized, aggregated GBD data, institutional review board approval was waived.

## 3 Results

### 3.1 Global patterns of thyroid cancer burden in males (2021)

**Figure 1:**
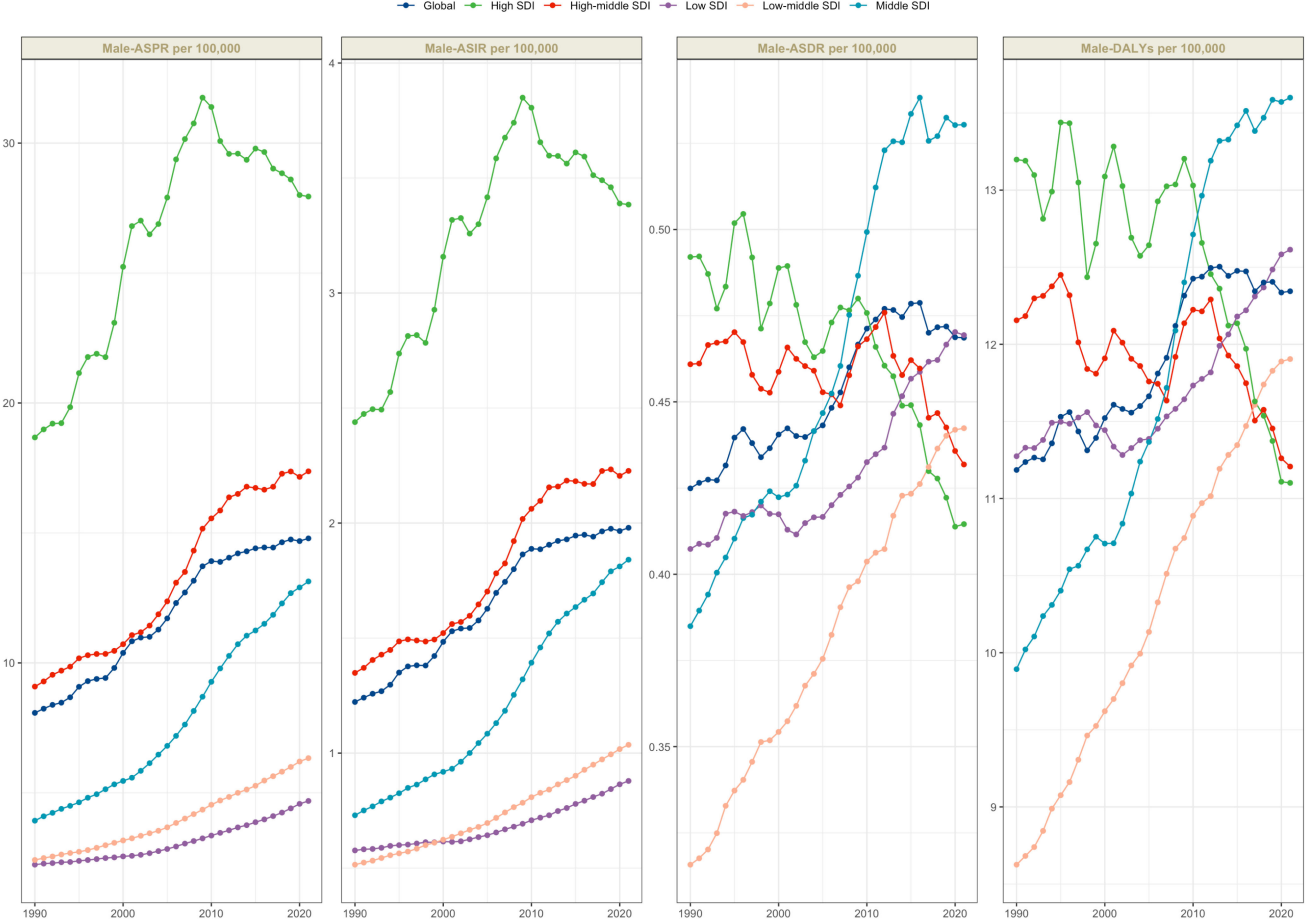
Trends in male thyroid cancer age-standardized prevalence rates, age-standardized incidence rates, age-standardized deaths rates and age-standardized disability-adjusted life years (DALYs) rates from 1990-2021.

In 2021, thyroid cancer posed a significant global health burden for males, with an estimated 82,301.4 (95% UI: 71,574.6–91,093) new cases (incidence), 627,658.1 (95% UI: 549,745–698,427.4) prevalent cases, 18,030.9 (95% UI: 15,238.3–19,895.3) deaths, and 504,440.4 (95% UI: 421,688.1–563,174.7) DALYs (**Tables 1**–**4**). The age-standardized rates (ASR) were 2.0 per 100,000 for incidence, 14.8 for prevalence, 0.5 for mortality, and 12.3 for DALYs (**Figure 1**).

**Table 1:** Prevalence of global and regional trends in male thyroid cancer burden.

Location	1990		2021		EAPC_95%CI
	Number	ASR	Number	ASR	
Global	180150.3 (172642.1-191834.7)	8.1 (7.7-8.6)	627658.1 (549745-698427.4)	14.8 (13-16.4)	2.23 (2.05 to 2.41)
High SDI	90401.7 (87702-93813.8)	18.7 (18.1-19.4)	217371.3 (207118.1-228095)	27.9 (26.6-29.3)	1.57 (1.22 to 1.92)
High-middle SDI	46754.3 (44063.3-50855.8)	9.1 (8.6-9.9)	150628.2 (128632-178366.5)	17.4 (14.9-20.6)	2.46 (2.27 to 2.64)
Middle SDI	28191 (24676.7-32433.8)	3.9 (3.5-4.5)	184907.2 (137943.8-219369.1)	13.1 (9.8-15.6)	4.35 (4.18 to 4.53)
Low-middle SDI	10725.7 (9442.3-12900.8)	2.4 (2.1-2.9)	55642.7 (46542.4-65475.9)	6.3 (5.3-7.4)	3.3 (3.22 to 3.37)
Low SDI	3853.9 (3171.4-4536.4)	2.2 (1.9-2.6)	18664.8 (13452.8-24270.9)	4.7 (3.4-6.1)	2.49 (2.34 to 2.65)
Andean Latin America	514.8 (429.6-602.6)	3.8 (3.1-4.4)	4096.4 (3203.3-5108.5)	12.9 (10.1-16.2)	4.25 (3.98 to 4.52)
Australasia	1517.7 (1340.8-1722.5)	13.9 (12.2-15.7)	6594.2 (5403.4-7862.2)	33 (26.8-39.6)	3.78 (3.47 to 4.09)
Caribbean	768.7 (703.3-846)	5.4 (4.9-5.9)	2988.9 (2552.8-3503.3)	11.6 (10-13.6)	2.69 (2.48 to 2.89)
Central Asia	1642.4 (1507.2-1802.8)	6.3 (5.7-6.9)	2777.7 (2396.8-3189.8)	6 (5.2-6.8)	-0.13 (-0.97 to 0.72)
Central Europe	8311.9 (7801.2-8813.2)	12.1 (11.3-12.8)	8936.4 (8042.4-9992.1)	10.8 (9.7-12)	-0.38 (-0.74 to -0.02)
Central Latin America	2563.2 (2429.5-2704.2)	4.5 (4.3-4.8)	14026.8 (12239.1-16069.5)	11.1 (9.7-12.7)	2.68 (2.53 to 2.82)
Central Sub-Saharan Africa	187 (138.5-248.9)	1.1 (0.8-1.5)	847.1 (541.5-1250.4)	1.9 (1.2-2.8)	1.75 (1.39 to 2.12)
East Asia	21622.4 (17483.8-28337.5)	3.8 (3-5)	161950.3 (116644.5-214942.1)	16 (11.6-21)	5.65 (5.31 to 6)
Eastern Europe	10967.5 (10348.3-12257.2)	9.3 (8.8-10.4)	19674.5 (17517-21821.6)	15.2 (13.6-16.8)	1.57 (1.36 to 1.79)
Eastern Sub-Saharan Africa	2126.1 (1624-2525.7)	3.6 (2.8-4.3)	11166.6 (7307.3-15313)	7.7 (5.1-10.5)	2.61 (2.4 to 2.82)
High-income Asia Pacific	10747 (10082.8-11586.4)	11 (10.4-11.9)	26766.1 (23650.2-32004.4)	17.5 (15.3-21.1)	2.02 (1.42 to 2.62)
High-income North America	37462.7 (36367.3-38462.7)	25.6 (24.8-26.3)	97605 (93268.4-102229.3)	38.6 (36.9-40.4)	1.32 (1.11 to 1.53)
North Africa and Middle East	6791.9 (5720.8-8572.8)	5.5 (4.7-6.9)	53696.8 (42631.6-62406.6)	17.3 (13.7-20)	4.21 (4.01 to 4.41)
Oceania	75.2 (52.3-100.9)	3 (2.1-4.1)	225.5 (145.3-318.1)	3.8 (2.4-5.3)	0.51 (0.33 to 0.69)
South Asia	10747.9 (9217.8-13374.2)	2.4 (2.1-3)	64142.7 (50360.7-77079.6)	7.1 (5.6-8.5)	3.79 (3.68 to 3.91)
Southeast Asia	8776.7 (7112.8-10069.5)	5.1 (4.2-5.9)	46383.6 (36301.6-55647.8)	12.4 (9.7-14.8)	2.84 (2.79 to 2.88)
Southern Latin America	1935.8 (1725.1-2163)	8.5 (7.6-9.6)	5106.5 (4456-5788.2)	13.6 (11.9-15.4)	1.68 (1.43 to 1.93)
Southern Sub-Saharan Africa	545.3 (453.2-639.8)	3 (2.5-3.6)	1926.2 (1530-2449.9)	5.4 (4.3-6.8)	1.8 (1.71 to 1.89)
Tropical Latin America	2689.2 (2544.2-2849.9)	4.6 (4.3-4.9)	11174.3 (10349.5-11943.8)	8.9 (8.3-9.5)	2 (1.83 to 2.18)
Western Europe	49871.8 (47045.2-53179.3)	22.2 (20.9-23.7)	86525 (78736-95356.7)	28 (25.5-30.8)	1.09 (0.51 to 1.67)
Western Sub-Saharan Africa	285.1 (221-349.2)	0.4 (0.3-0.5)	1047.5 (766.4-1356.3)	0.6 (0.4-0.8)	1.18 (1.04 to 1.31)

**Table 2:** Incidence of global and regional trends in male thyroid cancer burden.

Location	1990		2021		EAPC_95%CI
	Number	ASR	Number	ASR	
Global	25306.2 (24145.5-27281.6)	1.2 (1.2-1.3)	82301.4 (71574.6-91093)	2 (1.7-2.2)	1.78 (1.64 to 1.92)
High SDI	11682.8 (11338-12107)	2.4 (2.4-2.5)	27015.7 (25725.2-28391.1)	3.4 (3.2-3.6)	1.28 (0.98 to 1.59)
High-middle SDI	6484.9 (6105.5-7094.9)	1.3 (1.3-1.5)	19184 (16394.1-22647.4)	2.2 (1.9-2.6)	1.91 (1.75 to 2.07)
Middle SDI	4399.8 (3913.8-5052.4)	0.7 (0.7-0.9)	24857.4 (18575.3-29383)	1.8 (1.4-2.2)	3.34 (3.19 to 3.5)
Low-middle SDI	1909 (1674.6-2352.3)	0.5 (0.5-0.6)	8301 (7002.2-9659)	1 (0.9-1.2)	2.37 (2.32 to 2.42)
Low SDI	796.6 (653.3-932)	0.6 (0.5-0.7)	2884 (2105.6-3715.3)	0.9 (0.6-1.1)	1.37 (1.25 to 1.49)
Andean Latin America	93.5 (77.9-110.4)	0.8 (0.7-1)	586.4 (458.7-727.9)	1.9 (1.5-2.4)	2.96 (2.76 to 3.16)
Australasia	206.2 (183.4-231.5)	1.9 (1.7-2.1)	828.5 (685.8-978)	4 (3.3-4.8)	3.3 (3.02 to 3.58)
Caribbean	118.9 (109.8-129.2)	0.9 (0.8-1)	417.4 (357.1-484.9)	1.6 (1.4-1.9)	2.18 (1.99 to 2.38)
Central Asia	234.9 (216.3-257)	1 (0.9-1.1)	374.9 (324.8-425.7)	0.9 (0.8-1)	-0.4 (-1.18 to 0.39)
Central Europe	1253.5 (1184.2-1320.9)	1.9 (1.8-2)	1218.5 (1097.8-1355.2)	1.4 (1.3-1.6)	-0.91 (-1.3 to -0.53)
Central Latin America	426.9 (406.9-447.3)	0.9 (0.8-0.9)	2003.3 (1750.6-2290.6)	1.6 (1.4-1.9)	1.74 (1.57 to 1.92)
Central Sub-Saharan Africa	39.9 (29.9-53.4)	0.3 (0.2-0.4)	133.7 (85.8-193.4)	0.4 (0.3-0.6)	0.64 (0.37 to 0.91)
East Asia	3317.8 (2670.4-4369.5)	0.7 (0.6-0.9)	21005.9 (15298.9-27869.4)	2.1 (1.6-2.8)	4.36 (4.05 to 4.68)
Eastern Europe	1513.8 (1429.1-1687.3)	1.4 (1.3-1.5)	2621.3 (2334.1-2911.9)	2 (1.8-2.2)	1.24 (1.03 to 1.46)
Eastern Sub-Saharan Africa	456.9 (342.2-536.5)	1 (0.8-1.2)	1692.9 (1106.7-2290.5)	1.5 (1-2)	1.31 (1.16 to 1.45)
High-income Asia Pacific	1485.5 (1401-1594.5)	1.6 (1.5-1.7)	3649.7 (3247.4-4291.3)	2.2 (2-2.7)	1.44 (0.92 to 1.96)
High-income North America	4582.4 (4446.7-4703.8)	3.1 (3-3.2)	11820.5 (11294.5-12381.6)	4.6 (4.4-4.8)	1.22 (1.02 to 1.42)
North Africa and Middle East	877.7 (743.1-1125.6)	0.8 (0.7-1)	6345.3 (5068-7362.6)	2.1 (1.7-2.4)	3.71 (3.52 to 3.91)
Oceania	11.6 (8-15.6)	0.6 (0.4-0.8)	33.5 (21.2-46.5)	0.7 (0.4-0.9)	0.28 (0.11 to 0.45)
South Asia	1957.6 (1666.8-2474.2)	0.5 (0.5-0.7)	9620.6 (7670-11513.4)	1.2 (0.9-1.4)	2.64 (2.56 to 2.71)
Southeast Asia	1381.5 (1131.6-1596.6)	0.9 (0.8-1.1)	6430.7 (5006.8-7610.5)	1.8 (1.4-2.2)	2.17 (2.14 to 2.2)
Southern Latin America	309.9 (279.5-346.2)	1.4 (1.3-1.6)	710.7 (622-805.3)	1.9 (1.7-2.1)	1.06 (0.78 to 1.34)
Southern Sub-Saharan Africa	87.3 (71.5-103.6)	0.6 (0.5-0.7)	287.7 (233.1-355.7)	0.9 (0.8-1.1)	1.45 (1.33 to 1.56)
Tropical Latin America	430.8 (409.2-456.7)	0.8 (0.8-0.9)	1589.6 (1474-1695.8)	1.3 (1.2-1.4)	1.31 (1.16 to 1.47)
Western Europe	6466 (6122.3-6868.6)	2.9 (2.7-3)	10774.6 (9839.8-11847.7)	3.3 (3.1-3.7)	0.83 (0.31 to 1.35)
Western Sub-Saharan Africa	53.5 (41.2-64.8)	0.1 (0.1-0.1)	155.6 (116.6-202)	0.1 (0.1-0.1)	0.27 (0.16 to 0.39)

**Table 3:** Deaths of global and regional trends in male thyroid cancer burden.

Location	1990		2021		EAPC_95%CI
	Number	ASR	Number	ASR	
Global	7361.6 (6917.2-8196.8)	0.4 (0.4-0.5)	18030.9 (15238.3-19895.3)	0.5 (0.4-0.5)	0.41 (0.35 to 0.46)
High SDI	2237.3 (2160.4-2315.7)	0.5 (0.5-0.5)	3929.5 (3631.5-4144.6)	0.4 (0.4-0.4)	-0.5 (-0.6 to -0.4)
High-middle SDI	1859.1 (1737-2058)	0.5 (0.4-0.5)	3623.9 (3064.5-4202.4)	0.4 (0.4-0.5)	-0.12 (-0.2 to -0.05)
Middle SDI	1799.5 (1607.7-2122)	0.4 (0.3-0.5)	6294.4 (4699.4-7313)	0.5 (0.4-0.6)	1.21 (1.11 to 1.3)
Low-middle SDI	974.8 (845.1-1229.8)	0.3 (0.3-0.4)	2985.7 (2550.2-3451.8)	0.4 (0.4-0.5)	1.16 (1.12 to 1.19)
Low SDI	479.3 (389.5-555.4)	0.4 (0.3-0.5)	1182.6 (868.5-1500.9)	0.5 (0.3-0.6)	0.46 (0.39 to 0.53)
Andean Latin America	49.2 (40.6-58.4)	0.5 (0.4-0.6)	191.8 (148.7-241.4)	0.7 (0.5-0.9)	1.14 (0.97 to 1.3)
Australasia	39.9 (35.8-44.3)	0.4 (0.3-0.4)	101.1 (84.3-118)	0.4 (0.3-0.5)	0.74 (0.52 to 0.97)
Caribbean	47 (43.3-51.2)	0.4 (0.3-0.4)	127 (109.5-146.4)	0.5 (0.4-0.6)	1.08 (0.89 to 1.27)
Central Asia	88.9 (82.6-96.6)	0.4 (0.4-0.5)	112.1 (98.7-127.3)	0.3 (0.3-0.4)	-1.15 (-1.82 to -0.48)
Central Europe	464.6 (441.5-488.2)	0.7 (0.7-0.8)	325.7 (295.7-360)	0.4 (0.3-0.4)	-2.51 (-2.93 to -2.09)
Central Latin America	196.4 (188.9-204.6)	0.5 (0.5-0.5)	647.1 (566.6-739.1)	0.6 (0.5-0.7)	0.25 (0.06 to 0.45)
Central Sub-Saharan Africa	24.8 (18.6-33.5)	0.2 (0.2-0.3)	60 (38.8-86.3)	0.3 (0.2-0.4)	-0.02 (-0.2 to 0.17)
East Asia	1302.4 (1044.3-1714.3)	0.4 (0.3-0.5)	4400.1 (3042.4-5815.3)	0.5 (0.3-0.6)	1.18 (0.99 to 1.38)
Eastern Europe	396.5 (373.9-436.9)	0.4 (0.4-0.4)	537.3 (477.3-601.8)	0.4 (0.4-0.5)	-0.17 (-0.47 to 0.13)
Eastern Sub-Saharan Africa	284.9 (211.6-335)	0.7 (0.5-0.9)	688.8 (454-922.8)	0.8 (0.5-1.1)	0.41 (0.33 to 0.48)
High-income Asia Pacific	395.3 (374.4-428)	0.5 (0.5-0.5)	899 (811.1-1005)	0.4 (0.4-0.5)	-0.62 (-0.88 to -0.35)
High-income North America	563.2 (538-579.5)	0.4 (0.4-0.4)	1302.3 (1211.5-1370.3)	0.4 (0.4-0.5)	0.32 (0.19 to 0.45)
North Africa and Middle East	223.2 (188.1-304.3)	0.3 (0.2-0.4)	800.8 (669.5-915.8)	0.4 (0.3-0.4)	1.21 (1.07 to 1.34)
Oceania	4.5 (3.1-6.1)	0.3 (0.2-0.4)	11.9 (7.4-16.8)	0.3 (0.2-0.5)	0.02 (-0.1 to 0.15)
South Asia	1038.2 (876.7-1320)	0.3 (0.3-0.4)	3484.8 (2865.8-4119.1)	0.5 (0.4-0.6)	1.21 (1.16 to 1.25)
Southeast Asia	547.1 (461.5-628.6)	0.5 (0.4-0.5)	1797.9 (1390.3-2093.4)	0.6 (0.5-0.7)	0.9 (0.84 to 0.95)
Southern Latin America	126.5 (113.9-139.8)	0.6 (0.6-0.7)	199 (174.4-226.4)	0.5 (0.5-0.6)	-0.41 (-0.74 to -0.09)
Southern Sub-Saharan Africa	37.3 (29.7-45.7)	0.3 (0.2-0.4)	99.6 (83-116.6)	0.4 (0.3-0.5)	0.85 (0.65 to 1.05)
Tropical Latin America	187.5 (177.8-198.3)	0.5 (0.4-0.5)	515.5 (479.7-548.4)	0.5 (0.4-0.5)	0.1 (-0.05 to 0.25)
Western Europe	1314.1 (1255.3-1381.9)	0.6 (0.5-0.6)	1667.8 (1516.9-1806)	0.4 (0.4-0.4)	-0.97 (-1.13 to -0.82)
Western Sub-Saharan Africa	30 (22.8-35.8)	0.1 (0-0.1)	61.1 (46.8-81.8)	0.1 (0-0.1)	-0.56 (-0.68 to -0.45)

**Table 4:** DALYs of global and regional trends in male thyroid cancer burden.

Location	1990		2021		EAPC_95%CI
	Number	ASR	Number	ASR	
Global	225602 (210319.3-250805.8)	11.2 (10.5-12.5)	504440.4 (421688.1-563174.7)	12.3 (10.3-13.8)	0.41 (0.35 to 0.46)
High SDI	62540 (60110.3-65670.3)	13.2 (12.7-13.9)	96167.4 (89982.7-103224.4)	11.1 (10.4-11.9)	-0.46 (-0.59 to -0.34)
High-middle SDI	56387.5 (52409.4-62960.9)	12.2 (11.3-13.6)	97411.2 (82658.2-113994.8)	11.2 (9.5-13.1)	-0.2 (-0.27 to -0.13)
Middle SDI	58004.2 (51315.9-66743.5)	9.9 (8.8-11.6)	178242.8 (133247.3-206611.1)	13.6 (10.1-15.8)	1.19 (1.1 to 1.28)
Low-middle SDI	32177.7 (28012.5-40074.8)	8.6 (7.5-10.9)	91993.1 (77128.7-106758.1)	11.9 (10.1-13.8)	1.12 (1.09 to 1.14)
Low SDI	16149 (13018.5-18778.6)	11.3 (9.2-13.1)	40208.6 (29216.1-51848.8)	12.6 (9.3-16)	0.33 (0.27 to 0.39)
Andean Latin America	1497.4 (1248.4-1753.4)	12.9 (10.6-15.3)	5244.5 (4092.3-6554.3)	17.8 (13.9-22.3)	1.04 (0.87 to 1.21)
Australasia	1108.5 (988.8-1230.1)	10.3 (9.2-11.5)	2645.5 (2204.9-3081.1)	11.8 (9.9-13.9)	1.07 (0.83 to 1.32)
Caribbean	1376.5 (1258-1509.3)	10.3 (9.4-11.2)	3491.2 (2987.3-4022.1)	13.7 (11.8-15.8)	1.09 (0.9 to 1.28)
Central Asia	3072.4 (2851.5-3325.6)	13.3 (12.4-14.5)	3565.8 (3108.7-4065.9)	8.8 (7.7-10)	-1.52 (-2.17 to -0.86)
Central Europe	13791.4 (13066-14521.8)	20.6 (19.6-21.7)	8445.4 (7673.5-9281.1)	9.4 (8.6-10.4)	-2.67 (-3.09 to -2.24)
Central Latin America	6004.8 (5753.7-6256.4)	12.7 (12.2-13.2)	17893 (15698.3-20509)	15 (13.1-17.1)	0.27 (0.05 to 0.49)
Central Sub-Saharan Africa	822.1 (626.6-1084.9)	6.4 (4.7-8.6)	2086.5 (1357.3-3030.8)	6.4 (4.1-9.2)	-0.03 (-0.22 to 0.16)
East Asia	40821.6 (32428.8-53873.7)	9 (7.3-11.8)	114756.2 (79233.9-153071.8)	11.6 (8-15.3)	1.24 (1.05 to 1.43)
Eastern Europe	12864.4 (12133.1-14311.3)	11.8 (11.2-13.1)	15815.6 (13998.9-17704.7)	12 (10.6-13.4)	-0.24 (-0.55 to 0.08)
Eastern Sub-Saharan Africa	9644.1 (7068-11491.2)	20.1 (15-23.6)	24493.1 (16088.5-33150.7)	22.2 (14.6-29.8)	0.28 (0.19 to 0.38)
High-income Asia Pacific	10461.8 (9878.6-11434.5)	11.7 (11.1-12.8)	17780.9 (16219.4-20626.2)	9.4 (8.5-11)	-0.61 (-0.92 to -0.3)
High-income North America	16363.1 (15487.2-17237.4)	11.2 (10.6-11.8)	34361.1 (32175.2-36915.8)	12.5 (11.7-13.5)	0.28 (0.14 to 0.42)
North Africa and Middle East	7321.6 (6173.1-9558.1)	7.2 (6.1-9.7)	26384 (21578.2-30576.8)	10 (8.3-11.6)	1.39 (1.24 to 1.54)
Oceania	159.1 (108.5-213.5)	8.1 (5.5-11)	409.7 (251.3-579.9)	8.6 (5.3-12.2)	0.07 (-0.06 to 0.2)
South Asia	35050.8 (29910.3-44241.6)	9.5 (8-12)	107516.5 (85892.4-128100)	13.3 (10.8-15.8)	1.16 (1.13 to 1.2)
Southeast Asia	17521.6 (14396.8-20199)	12.4 (10.5-14.3)	54459.7 (41138-64516.6)	16.3 (12.6-19.2)	0.9 (0.86 to 0.95)
Southern Latin America	3616.9 (3286.3-3968.2)	16.8 (15.3-18.5)	5185.8 (4577.3-5867.6)	13.7 (12.1-15.5)	-0.55 (-0.87 to -0.22)
Southern Sub-Saharan Africa	1278.8 (1038.2-1545.3)	8.6 (6.8-10.5)	3370 (2752.9-4043.4)	11.4 (9.4-13.4)	0.81 (0.59 to 1.02)
Tropical Latin America	5934.3 (5630.7-6272.6)	11.9 (11.3-12.6)	14346 (13396-15266.8)	12.1 (11.3-12.9)	-0.02 (-0.16 to 0.12)
Western Europe	35821.8 (34069.1-38065.4)	15.4 (14.6-16.4)	39789.3 (36319.9-43515.3)	10.9 (9.9-11.9)	-0.94 (-1.18 to -0.7)
Western Sub-Saharan Africa	1069 (834.9-1283.3)	1.8 (1.3-2.1)	2400.4 (1775.4-3087.4)	1.7 (1.3-2.2)	-0.38 (-0.48 to -0.27)

### 3.2 Socio-demographic index stratification

**Figure 2:**
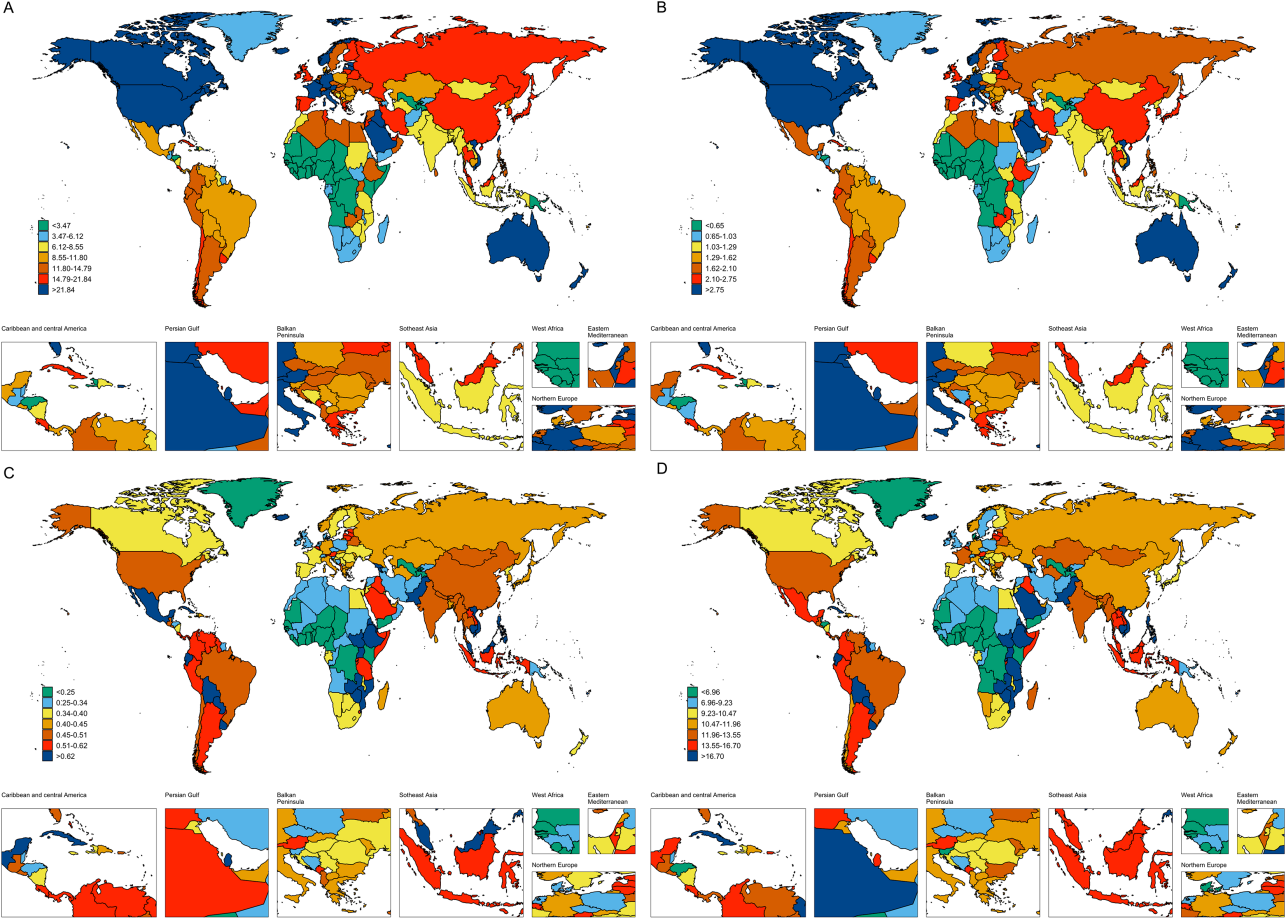
The age-standardized prevalence rates **(A)**, age-standardized incidence rates **(B)**, age-standardized deaths rates **(C)** and age-standardized disability-adjusted life years (DALYs) rates **(D)** of male thyroid cancer in 204 countries and territories.

A profound socio-economic gradient was observed for incidence and prevalence. The High SDI region exhibited the highest ASIR (3.4) and ASPR (27.9), which were approximately 3.8-fold and 6-fold higher than those in the Low SDI region (ASIR: 0.9; ASPR: 4.7), respectively. Geographically, High-income North America (ASIR: 4.6; ASPR: 38.6) and Western Europe (ASIR: 3.3; ASPR: 28.0) bore the highest burden of new and existing cases (**Figures 2A, B**). In stark contrast, the mortality and DALY rates showed a flattened or even inverted gradient (**Figures 2C, D**). The Low SDI region had a mortality rate (0.5) comparable to the High SDI region (0.4), while Eastern Sub-Saharan Africa suffered the highest DALYs rate (22.2), indicating worse outcomes in less developed regions (**Table 4**).

### 3.3 Temporal trends: the incongruence between diagnosis and outcome

**Figure 3:**
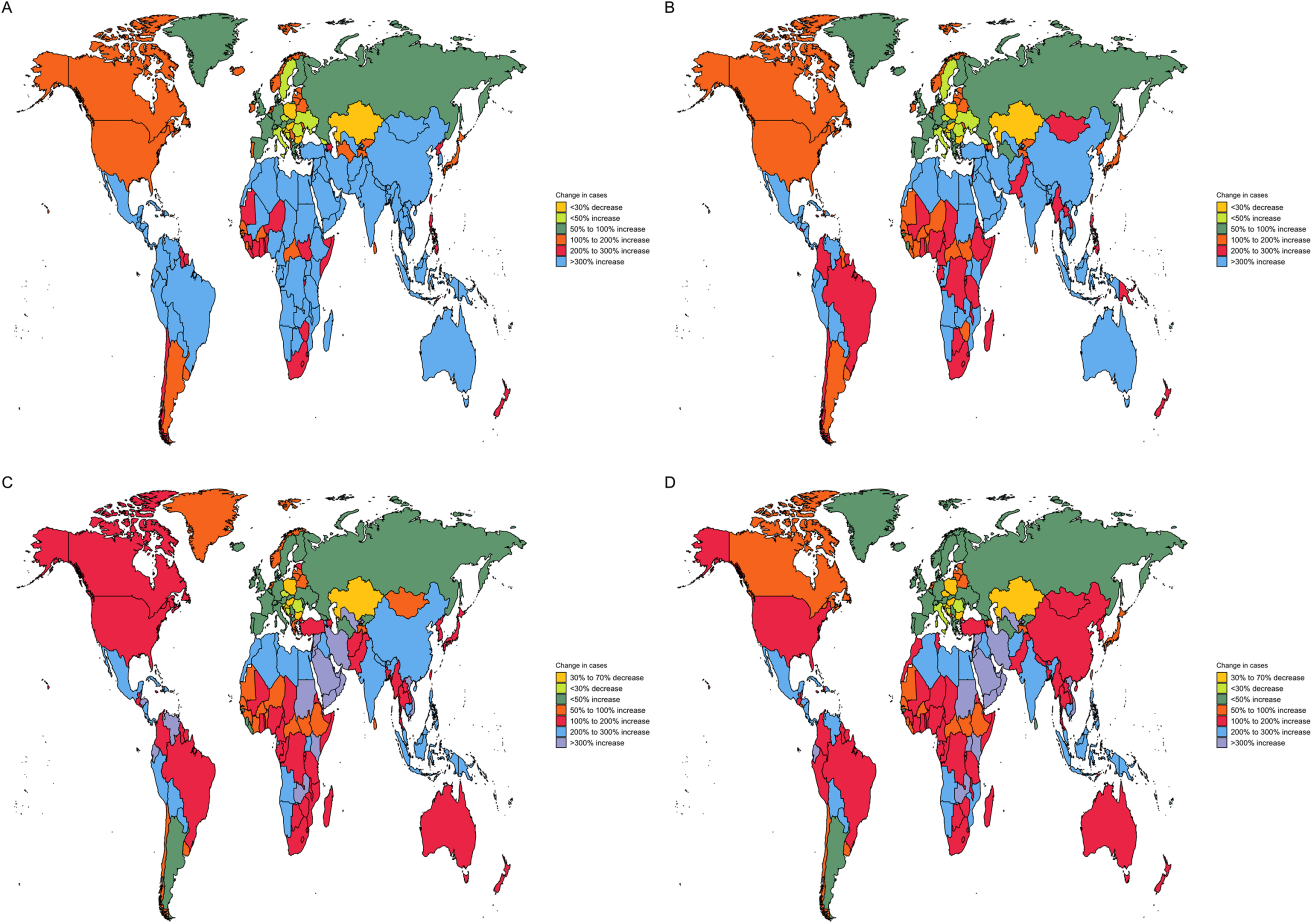
Changes prevalence cases **(A)**, incidence cases **(B)**, deaths cases **(C)** and disability-adjusted life years (DALYs) **(D)** of male thyroid cancer in 204 countries and territories.

From 1990 to 2021, the global number of incident cases more than tripled, and prevalent cases increased by 3.5-fold. The age-standardized incidence rate (ASIR) exhibited a significant increase, with an EAPC of 1.78% (95% CI: 1.64 to 1.92), rising from 1.2 to 2.0 per 100,000 (**Table 2**). The ASPR increased even more substantially, from 8.1 to 14.8 (EAPC: 2.23%) (**Table 1**). This surge in diagnosis was overwhelmingly driven by certain regions. East Asia experienced the most dramatic rise, with its ASIR increasing by 200% (EAPC: 4.36%) and its number of incident cases soaring by over 600% (**Figures 3A, B**). Similarly, the North Africa and Middle East region (EAPC of ASIR: 3.71%) and Middle SDI quintile (EAPC of ASIR: 3.34%) showed remarkably high growth rates. In contrast, Central Europe (EAPC of ASIR: -0.91%) and Central Asia (EAPC of ASIR: -0.40%) demonstrated stable or declining incidence trends (**Table 2**).

Strikingly, this explosive growth in new diagnoses was decoupled from mortality outcomes. Globally, the age-standardized deaths rates (ASDR) remained largely stable, with a minimal EAPC of 0.41% (**Table 3**). Even in regions with skyrocketing incidence, mortality increases were modest at best (e.g., East Asia EAPC for mortality: 1.18%). Most notably, many high-SDI regions, including Central Europe (EAPC: -2.51%) and Western Europe (EAPC: -0.97%), achieved significant reductions in mortality despite high incidence (**Figure 3C**).

### 3.4 The evolving burden of disease

The trend in DALYs, which synthesizes fatal and non-fatal outcomes, revealed a global bifurcation in the disease burden. The global age-standardized DALYs rate saw a minimal net increase (EAPC: 0.41%), but this masked divergent regional pathways. Regions such as Central Europe (EAPC: -2.67%), Western Europe (EAPC: -0.94%), and High-income Asia Pacific (EAPC: -0.61%) demonstrated significant declines in DALYs rates (**Table 4**). In contrast, several low- and middle-SDI regions, including the North Africa and Middle East (EAPC: 1.39%), South Asia (EAPC: 1.16%), and the Middle SDI quintile (EAPC: 1.19%), exhibited significant increases in their DALYs rates (**Figure 3D**). This suggests that these regions are now facing the dual challenge of rising diagnosis (leading to increases in years lived with disability) without having yet fully attained the mortality reductions seen in higher-SDI areas, resulting in a growing overall burden.

### 3.5 Burden of thyroid cancer associated with high body-mass index in males

**Figure 4:**
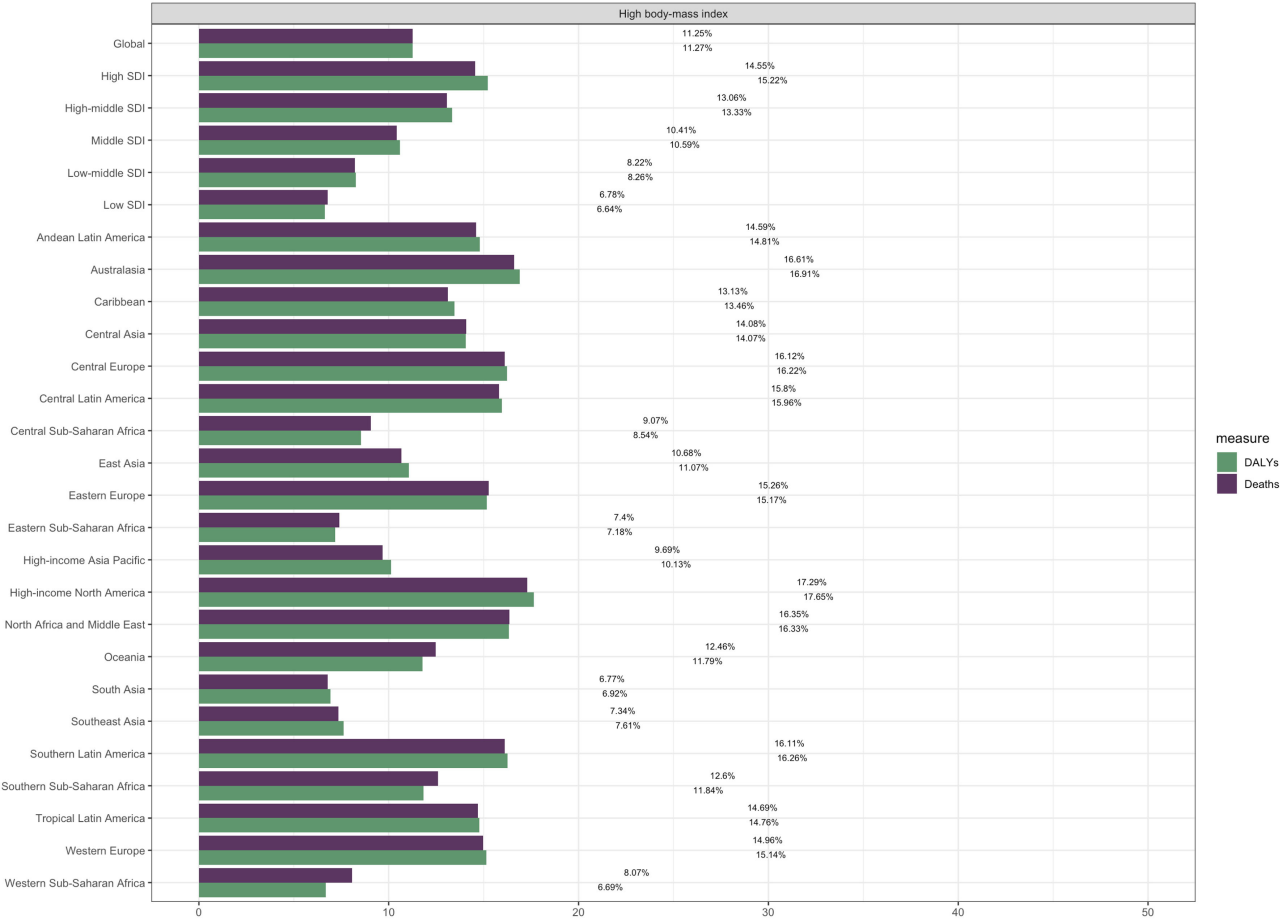
Attributable fractions of high body-mass index for thyroid cancer-related deaths and DALYs.

Globally, high body-mass index (BMI) was associated with 11.25% (95% UI: 8.48–14.12) of thyroid cancer deaths and 11.27% (95% UI: 8.56–14.17) of thyroid cancer DALYs among males in 2021 (**Table 4**). Significant regional variations were observed, with the highest attributable fractions in high-income regions. High-income North America showed the highest proportion of deaths linked to high BMI (17.29%, 95% UI: 13.22–21.10), followed by Australasia (16.61%, 95% UI: 12.58–20.68) and Southern Latin America (16.11%, 95% UI: 12.16–19.97) (**Figure 4**).

A clear socio-demographic gradient was evident, with High-SDI regions having the highest proportion of deaths associated with high BMI (14.55%, 95% UI: 11.03–17.96) and low-SDI regions the lowest (6.78%, 95% UI: 5.16–8.67). This pattern was consistent for DALYs, reflecting the global distribution of obesity prevalence.

### 3.6 Age-specific patterns and projections

**Figure 5:**
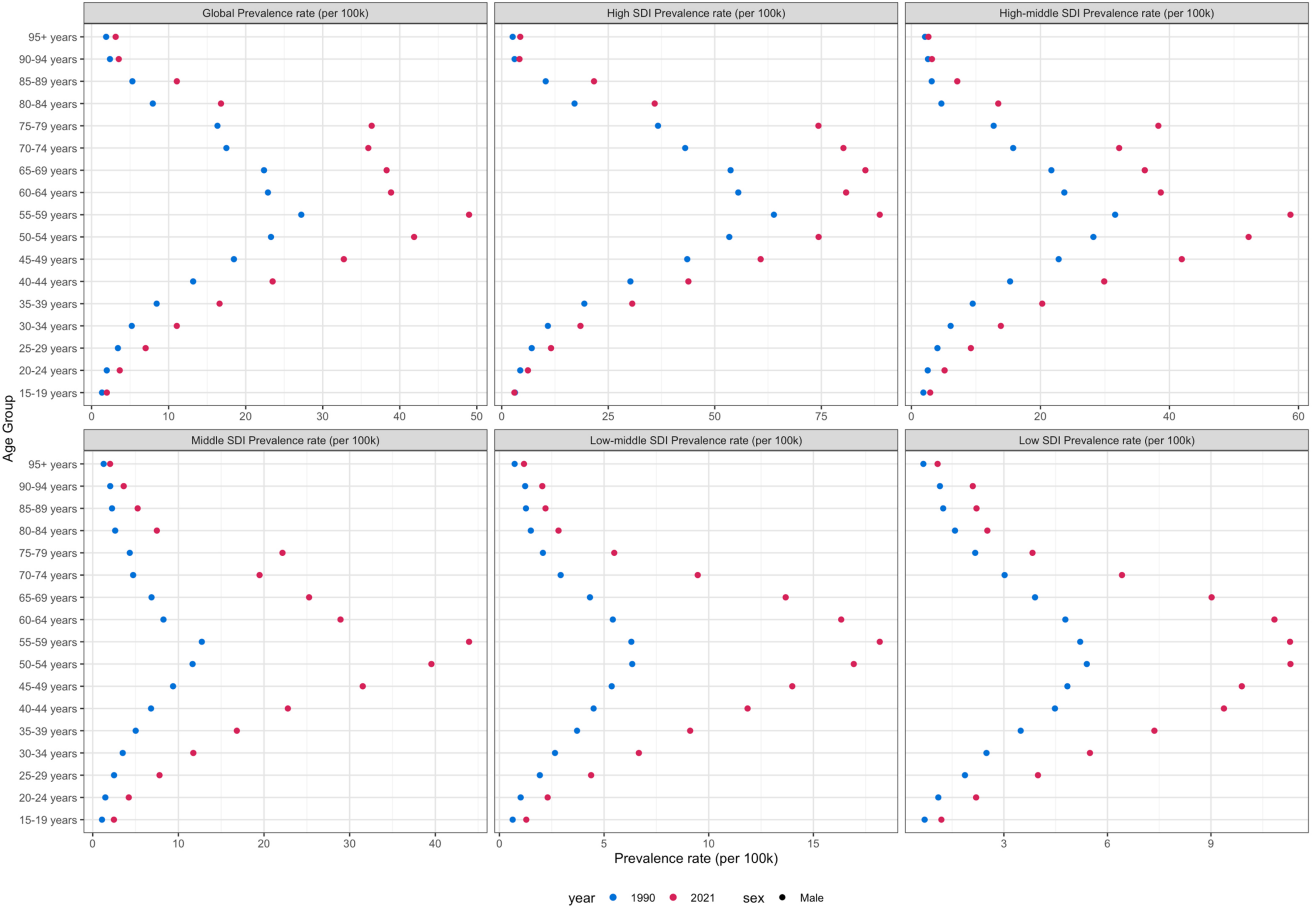
Age-standardized prevalence rates of male thyroid cancer by age group, socio-demographic index, 1990 and 2021.

**Figure 6:**
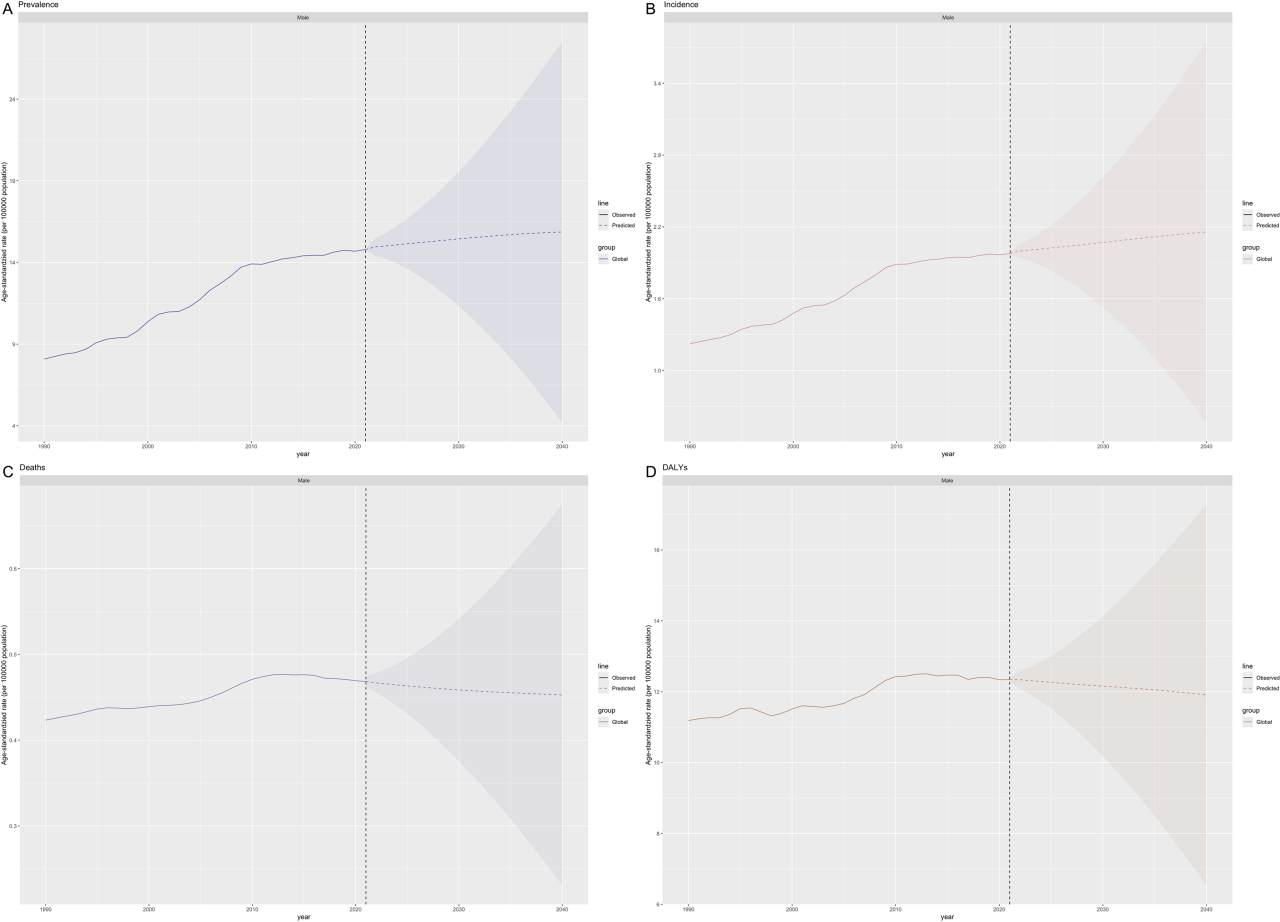
Future forecasts of age-standardized prevalence rates **(A)**, age-standardized incidence rates **(B)**, age-standardized deaths rates **(C)** and age-standardized disability-adjusted life years (DALYs) rates **(D)** of male thyroid cancer.

ASPR peaked at ages 55–59 years globally in both 1990 and 2021. Low-SDI regions shifted from a peak at 50–54 years (1990) to 55–59 years (2021), while other SDI regions consistently peaked at 55–59 years (**Figure 5**). The Bayesian Age-Period-Cohort (BAPC) model demonstrated excellent predictive performance in validation tests using 1990–2015 data to predict 2016–2021 outcomes. For prevalence, the model achieved a mean absolute percentage error (MAPE) of 1.21%. For incidence, the MAPE was 0.41%. Mortality predictions showed a MAPE of 1.07%, and DALYs predictions achieved a MAPE of 1.13% (**Supplementary Table 1**). Based on this validated model, the age-standardized prevalence rate of thyroid cancer in males is projected to increase from 14.79 per 100,000 in 2021 to 15.70 in 2035 (6.2% increase), reaching 15.87 by 2040 (**Figure 6A**). The age-standardized incidence rate is projected to show a gradual increase from 1.98 per 100,000 in 2021 to 2.12 in 2035, and further to 2.16 in 2040, representing a 9.1% increase over the 19-year period (**Figure 6B**). In contrast, the age-standardized mortality rate is projected to remain relatively stable, decreasing slightly from 0.47 to 0.46 per 100,000 by 2040 (**Figure 6C**). The age-standardized DALY rate is projected to decrease moderately from 12.34 to 11.91 per 100,000 by 2040 (3.5% decline) (**Figure 6D**). All projections show progressively widening uncertainty intervals over time, reflecting increasing uncertainty in long-term predictions. By 2040, the UI for prevalence expands to 6.54 - 17.28, for incidence to 0.57 - 3.75, for mortality to 0.23 - 0.67, and for DALYs to 6.54 - 17.28.

## 4 Discussion

This comprehensive analysis of the global burden of thyroid cancer in males from 1990 to 2021, with projections to 2040, reveals several noteworthy epidemiological patterns that warrant careful consideration in both clinical and public health contexts. The findings demonstrate a complex interplay between increasing diagnostic intensity, changing risk factors, and evolving clinical practices across different world regions.

The most striking finding is the dissociation between incidence and mortality trends. While age-standardized incidence rates (ASIR) increased substantially (EAPC = 1.78%) between 1990 and 2021, age-standardized death rates (ASDR) remained largely stable (EAPC = 0.41%). This pattern is particularly evident in regions experiencing rapid incidence increases, such as East Asia (EAPC = 4.36%) and North Africa and Middle East (EAPC = 3.71%). While this dissociation is consistent with the phenomenon of overdiagnosis driven by advanced imaging technologies, we acknowledge that multiple factors likely contribute to this pattern (25, 26). The observed trends could reflect not only the detection of indolent tumors but also genuine improvements in treatment efficacy, enhanced healthcare access leading to earlier diagnosis of consequential cancers, or changes in disease classification systems. The relative contribution of these factors likely varies across healthcare systems and regions, and the ecological study design cannot definitively quantify their individual impacts.

The geographical variations observed reveal critical disparities that merit particular emphasis. East Asia’s dramatic rise in incidence (over 600% increase in case numbers) coincides with rapid healthcare modernization and widespread adoption of diagnostic technologies in the region. Conversely, Sub-Saharan Africa’s disproportionately high DALY burden despite lower incidence rates highlights the persistent challenges in treatment access and late-stage diagnosis in resource-limited settings. The inverted socio-economic gradient observed for mortality and DALYs, where low-SDI regions experience comparable or worse outcomes despite lower incidence, underscores the urgent need to improve treatment accessibility alongside optimizing diagnostic practices. Regions experiencing rapid increases in incidence may benefit from implementing more conservative diagnostic strategies, similar to approaches adopted in some high-SDI regions that have maintained excellent survival outcomes while addressing potential overdiagnosis (15, 27).

A substantial proportion of male thyroid cancer burden was associated with high body-mass index (11.25% of deaths and 11.27% of DALYs globally). It is crucial to emphasize that this association, while statistically significant and biologically plausible, does not establish causation. The observed socio-demographic gradient, with high-SDI regions bearing the greatest obesity-associated burden, aligns with global obesity epidemiology patterns (28, 29). The mechanisms underlying this association may involve insulin resistance, chronic inflammation, and adipokine-mediated pathways that promote thyroid cell proliferation and carcinogenesis (30, 31).

The concentration of this associated burden in middle-aged males (peaking at 55–59 years) may reflect cumulative exposure to obesity-related metabolic disturbances (32, 33). The analysis focused on high BMI due to its established association with thyroid cancer and data availability within the GBD framework. Other important risk factors, including radiation exposure, iodine intake, and endocrine disruptors, were not included due to limitations in global, gender-specific data availability.

The regional variations in attributable burden reflect both differences in obesity prevalence and variations in background thyroid cancer rates. High-income regions, particularly North America and Australasia, showed the highest proportions of thyroid cancer burden associated with high BMI (16-18%), aligning with their high obesity rates (34, 35). This pattern suggests that obesity may be contributing to the increasing thyroid cancer incidence observed in these regions, beyond the well-documented effects of diagnostic intensity.

The lower attributable fractions in low-SDI regions (approximately 7%) likely reflect both lower obesity prevalence and competing risks from other health conditions in these populations (3, 36). However, as these regions undergo nutritional transitions and obesity rates rise, the proportion of thyroid cancer associated with high BMI may increase accordingly (37). This trend underscores the need for preventive strategies that address the growing obesity epidemic in developing regions.

The projected trends to 2040, validated through rigorous back-testing, suggest a continuing increase in prevalence rates, indicating a growing population of male thyroid cancer survivors who will require long-term monitoring and care (38). However, the stable mortality projections across all SDI regions indicate that these cases are largely manageable with current treatment approaches when accessible. The robust performance of Bayesian Age-Period-Cohort (BAPC) model in validation tests (MAPE range: 0.41-1.21% across all indicators) provides strong support for reliability of the projections. The substantial widening of uncertainty intervals underscores the need for cautious interpretation of long-term predictions and highlights the potential impact of changing clinical practices on disease burden estimates.

The findings have important clinical and public health implications. First, they suggest that weight management interventions could potentially reduce a significant proportion of thyroid cancer burden, particularly in high-income countries. Second, the age distribution of the attributable burden highlights middle-aged men as a priority population for targeted interventions. Finally, the regional variations emphasize the need for gender-specific approaches that consider local patterns of obesity and thyroid cancer incidence.

Several important limitations should be considered when interpreting the findings. First, the GBD estimates rely on available vital registration and cancer registry data, which may be incomplete in some regions, particularly in low-SDI countries (18). Second, the projections assume continuation of current trends and do not account for potential changes in diagnostic guidelines or treatment paradigms that might significantly alter future disease patterns (15, 27). Third, the widening uncertainty intervals in later years reflect the inherent challenges in long-term forecasting of cancer burden, especially for malignancies like thyroid cancer where diagnostic practices significantly influence incidence patterns (39).

## 5 Conclusion

The findings highlight critical geographical disparities, with high-SDI regions experiencing stable trends due to refined diagnostic approaches, while middle-SDI regions show rapid increases as they adopt intensive screening methods. These patterns emphasize the need for region-specific strategies that balance early detection with avoidance of overdiagnosis.

The projections suggest continued incidence growth with stable mortality through 2040, though widening uncertainty intervals underscore the need for cautious interpretation. Healthcare systems should implement evidence-based, sex-specific approaches that prioritize appropriate diagnosis and management rather than indiscriminate detection. Future efforts should focus on developing gender-sensitive clinical guidelines, improving risk stratification tools for male patients, and translating increased detection into meaningful outcome improvements.

## Data Availability

The original contributions presented in the study are included in the article/**Supplementary Material**. Further inquiries can be directed to the corresponding author.
