# Supplementary material for: Global burden of thyroid cancer in males: a comprehensive analysis of incidence, mortality, and risk factors from 1990 to 2040
Source: Front Oncol. 2026 Feb 19;16:1699986. doi: 10.3389/fonc.2026.1699986 (PMC12960184; doi:10.3389/fonc.2026.1699986)
Supplement: Supplementary file 1 [file Table1.docx]

Supplementary Table 1 Performance Metrics of Bayesian Age-Period-Cohort (BAPC) Model in Predicting Thyroid Cancer Burden among Males

| Indicator | Training Period | Validation Period | MAPE (%) | MAE | RMSE |
| --- | --- | --- | --- | --- | --- |
| Prevalence | 1990-2015 | 2016-2021 | 1.21 | 0.177 | 0.197 |
| Incidence | 1990-2015 | 2016-2021 | 0.41 | 0.008 | 0.010 |
| Death | 1990-2015 | 2016-2021 | 1.07 | 0.005 | 0.006 |
| DALYs | 1990-2015 | 2016-2021 | 1.13 | 0.140 | 0.153 |

MAPE: mean absolute percentage error; MAE: mean absolute error; RMSE: root mean square error.
